# Protective Effects of Scutellarin on Human Cardiac Microvascular Endothelial Cells against Hypoxia-Reoxygenation Injury and Its Possible Target-Related Proteins

**DOI:** 10.1155/2015/278014

**Published:** 2015-10-18

**Authors:** Meina Shi, Yingting Liu, Lixing Feng, Yingbo Cui, Yajuan Chen, Peng Wang, Wenjuan Wu, Chen Chen, Xuan Liu, Weimin Yang

**Affiliations:** ^1^School of Pharmaceutical Science & Yunnan Key Laboratory of Pharmacology for Natural Products, Kunming Medical University, Kunming 650500, China; ^2^Shanghai Institute of Materia Medica, Chinese Academy of Sciences, Shanghai 201203, China; ^3^Department of Periodontology and Implant Dentistry, The First People's Hospital of Yun-Nan Province, Kunhua Hospital, Kunming University of Science and Technology, Kunming 650032, China

## Abstract

Scutellarin (SCU) is one of the main components of traditional Chinese medicine plant *Erigeron breviscapus (Vant.)* Hand.-Mazz. In this paper, we studied the protective effects of SCU on human cardiac microvascular endothelial cells (HCMECs) against hypoxia-reoxygenation (HR) injury and its possible target-related proteins. Results of MTT assay showed that pretreatment of SCU at doses of 1, 5, and 10 *μ*M for 2 h could significantly inhibit the decrease in cell viability of HCMECs induced by HR injury. Subcellular fractions of cells treated with vehicle control, 1 *μ*M SCU, HR injury, or 1 *μ*M SCU + HR injury were separated by ultracentrifugation. The protein expression profiles of cytoplasm and membrane/nuclei fractions were checked using protein two-dimensional electrophoresis (2-DE). Proteins differentially expressed between control and SCU-treated group, control and HR group, or HR and SCU + HR group were identified using mass spectrometry (MS/MS). Possible interaction network of these target-related proteins was predicted using bioinformatic analysis. The influence of SCU on the expression levels of these proteins was confirmed using Western blotting assay. The results indicated that proteins such as p27BBP protein (EIF6), heat shock 60 kDa protein 1 (HSPD1), and chaperonin containing TCP1 subunit 6A isoform (CCT6A) might play important roles in the effects of SCU.

## 1. Introduction

SCU (4,5,6-trihydroxyflavone-7-glucuronide) is a flavone isolated from* Erigeron breviscapus (Vant.)* Hand.-Mazz. Extract containing mainly SCU, called as breviscapine, has been successfully used in China in clinic for treatment of both cardiac ischemic and cerebral ischemic diseases [1–5]. Previous studies showed that purified SCU had protective effects on cardiovascular ischemia as well as cerebrovascular diseases and its effects were better than those of breviscapine [5]. SCU was found to have protective effects on cardiomyocytes against ischemic injury [6, 7]. Endothelium was an active early participant in ischemia-reperfusion (IR) injury and endothelial cells were particularly susceptible to and actively participate in IR injury [8–10]. In the present study, we checked whether SCU could also protect endothelial cells against simulated IR injury, 12 h hypoxia followed by 12 h reoxygenation. Firstly, we observed the effects of SCU on cell viability of HCMECs under normal condition. Then, protective effects of SCU on cell viability of HCMECs underwent HR treatment were studied.

For a comprehensive study of the mechanism of SCU on HCMECs, a proteomic approach (2-DE combined with MS/MS) was used for seeking possible target-related proteins of SCU in HCMECs, both under normal condition and under HR treatment. Proteins differentially expressed between control and SCU-treated group and proteins differentially expressed between HR and SCU + HR group were considered as possible target-related proteins of SCU. At the same time, proteins differentially expressed between control and HR group were also identified to provide information about possible proteins involved in HR injury. Using proteomic approach offers us the opportunity to search possible target-related proteins of SCU in an unbiased way. Furthermore, subcellular fractions of cells were separated and used in proteomic analysis to increase the coverage of analysis and study possible cross-talk between different cell organelles.

## 2. Materials and Methods

### 2.1. Reagents

**Figure 1:**
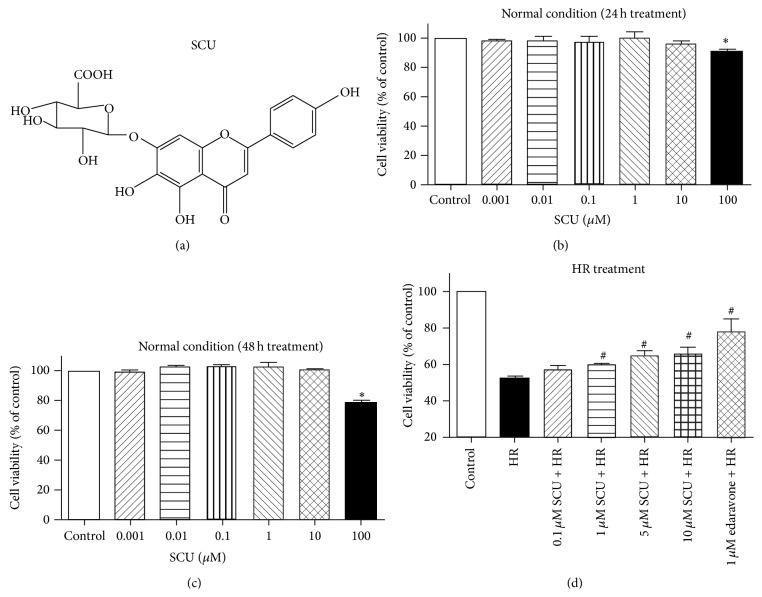
Effects of SCU on cell viability of HCMECs under normal condition or HR treatment. (a) Chemical structure of SCU. (b) Results of MTT assay of cell viability of cells treated with SCU at different concentrations or vehicle control for 24 h. ^*∗*^ *p* < 0.05, compared with control. (c) Results of MTT assay of cell viability of cells treated with SCU at different concentrations or vehicle control for 48 h. ^*∗*^ *p* < 0.05, compared with control. (d) Results of MTT assay of cell viability of cells treated with vehicle control, HR treatment, and HR treatment with 2 h pretreatment of SCU at different concentrations. ^#^ *p* < 0.05, compared to HR group. Data are presented as mean ± SD, *n* = 3 independent experiments.

Powdered SCU (purity 99%, formula weight 464.4) was obtained from Mr. Renwei Zhang of Kunming Longjin Pharmaceuticals Co. (Kunming, China). The structure of SCU was shown in Figure 1(a). SCU was dissolved in physiological saline solution. Cell culture reagents including modified RPMI-1640 medium and fetal bovine serum were obtained from HyClone (Thermo Fisher Scientific, Waltham, MA, USA). Edaravone with a purity of 99% was purchased from Sigma-Aldrich Chemical Co. (St. Louis, MO, USA).

### 2.2. Cell Culture

HCMECs were obtained from the Shanghai Yansheng Biochemical Technology Company (Shanghai, China) and grown in 1640 medium supplemented with 10% fetal bovine serum and 1% penicillin/streptomycin antibiotics. Tightly confluent monolayers of HCMECs from the 4th to the 15th passages were used in all experiments.

### 2.3. Effects of SCU on Cell Viability under Normal Condition or HR Treatment

Cell viability was determined using MTT assay. To check the influence of SCU on cell viability under normal condition, cells were plated in 96-well flat-bottomed plates at density of 3 × 10^3^ cells/well in complete medium and incubated overnight. Then, cells were treated with vehicle control (physiological saline solution) or SCU with final concentrations of 1 nM, 10 nM, 100 nM, 1 *μ*M, 10 *μ*M, and 100 *μ*M for 24 or 48 h. At the end of the incubation, 20 *μ*L of the dye (3, [4,5-dimethylthiazol-2-yl-] diphenyltetrazolium bromide, 5 mg/mL), MTT, was added to each well and the plates were incubated for 3 h at 37°C. Then, 100 *μ*L of lysis buffer (20% sodium dodecyl sulfate [SDS] in 50% N,N-dimethylformamide, containing 0.5% [v : v] 80% acetic acid and 0.4% [v : v] 1 N HCL), was added to each well and incubated overnight (16 h). Cell viability was evaluated by measuring the mitochondrial-dependent conversion of the yellow tetrazolium salt MTT to purple formazan crystals by metabolic active cells. The optical density (proportional to the number of live cells) was assessed with a Microplate Reader Bio-Rad 550 at 570 nm. Each experiment was performed in triplicate. Results of three independent experiments were used for statistical analysis.

To check the influence of SCU on cell viability under HR treatment, cells were plated in 96-well flat-bottomed plates at a density of 3 × 10^3^ cells/well in complete medium and incubated overnight. HR injury was induced as described before with minor modifications [11]. Briefly, cells were placed in a humidified hypoxic chamber (HF100, Heal Force Biotech Co., Shanghai, China) for 12 h of hypoxia (5% CO_2_ + 2% O_2_ + 93% N_2_) with medium free of glucose and serum at 37°C, followed by 12 h of reoxygenation (5% CO_2_ + 95% air) in complete medium containing glucose and serum. Cells were classified into four groups, control, HR, SCU + HR, and edaravone + HR group. Cells of HR group were pretreated with vehicle control (physiological saline solution) for 2 h and then underwent HR treatment. Cells of SCU + HR group were treated with SCU at nontoxic doses, with final concentrations of 8 nM, 40 nM, 100 nM, 200 nM, 1 *μ*M, 5 *μ*M, 10 *μ*M, and 25 *μ*M, for 2 h and then underwent HR treatment. Cells of edaravone + HR group (positive control group) were treated with edaravone with final concentration of 100 nM for 2 h and then underwent HR treatment. Control cells were cultured in parallel and kept in normal culture condition for the entire time period (26 h). After treatments, cell viability of different groups was determined using MTT assay as described above.

### 2.4. Proteomic Analysis

To make sure correspondence between the reaction of cells in experiment of checking cell viability using MTT assay and the reaction of cells in proteomic study, cells were plated in 96-well plates and 75 cm^2^ flasks at similar density and used for MTT assay and proteomic study, respectively. Only after MTT assay of cells in 96-well plates confirmed the protective effects of SCU on cells against HR injury, cells collected from 75 cm^2^ flasks were further used for proteomic study. Cells were classified into 4 groups, control, HR, SCU (1 *μ*M), and SCU + HR (SCU at 1 *μ*M) groups. After treatments, cells in 75 cm^2^ flasks were washed three times with ice-cold PBS and then scraped off with a cell scraper. After centrifugation at 2500 ×g for 3 min, the supernatants were discarded and cell pellets were resuspended for 5 min on ice in 500 *μ*L CLB buffer containing 10 mM HEPES, 10 mM NaCL, 1 mM KH_2_PO_4_, 5 mM NaHCO_3_, 5 mM EDTA, 1 mM CaCL_2_, and 0.5 mM MgCL_2_. Homogenization of the cells was achieved by ultrasonication (30 strokes, 4 HZ) on ice. Thereafter, 50 *μ*L of 2.5 M sucrose was added to restore isotonic conditions before differential centrifugation at 4°C. The first round of centrifugation was performed at 6500 g for 5 min. The pellet was supposed to contain nuclei. The supernatant was further ultracentrifugated at 107000 g for 30 min in a Hitachi CS150GXL ultracentrifuge. The resulting supernatant was supposed to contain cytoplasm and the pellet was supposed to contain membrane fraction. Then, the purity of the cytoplasm fraction, nuclei fraction, and membrane fraction was checked with Western blotting assay using antibodies against autophagy-related protein 7 (ATG7), histones (H3), and sodium-potassium adenosine triphosphatase (AT1A1) which were specific for cytoplasm, nuclei, and membrane, respectively. Since the results showed that the purity of cytoplasm fraction was acceptable while the membrane fraction contained part of nuclei fraction, the cytoplasm fraction was used without further treatment for proteomic analysis while the membrane fraction and nuclei fraction were merged together to be nuclei/membrane fraction. Protein samples from at least three independent experiments were collected and used in proteomic analysis.

Protein two-dimensional electrophoresis (2-DE) was conducted as reported in our previous studies [12–14]. The nuclei/membrane fraction were dissolved in 2-DE lysis buffer containing 7 M urea, 2 M thiourea, 2% CHAPS, 1% DTT, and 0.8% pharmalyte and protease inhibitor (all from Bio-Rad) on ice for 30 min. After centrifugation at 15000 ×g for 30 min at 4°C, the supernatant containing solubilized nuclei/membrane proteins could be used for 2-DE analysis. The proteins in cytoplasm fraction were precipitated by adding 2X volume of precooled acetone. After centrifugation at 15000 ×g for 15 min at 4°C, the pellet was washed twice with 75% ethanol, dried down by evaporation in a vacuum concentrator/centrifugal evaporator, and then redissolved in 2-DE lysis buffer for 2-DE use. The protein concentrations of cytoplasm fraction or nuclei/membrane fraction were determined using the Bradford method.

For 2-DE analysis, 150 *μ*g protein sample was applied for IEF using the ReadyStrip IPG Strips, 17 cm, and pH 4–7 (Bio-Rad). The strips were placed into a Protein IEF cell (Bio-Rad) and were rehydrated at 50 V for 12 h and then the proteins were separated based on their pI according to the following protocol: 250 V with linear climb for 30 min, 1000 V with rapid climb for 60 min, 10000 V with linear climb for 5 h, and 10000 V with rapid climb until 60 000 Vh was reached. After IEF, the IPG strips were equilibrated and then directly applied onto 12% homogeneous SDS-PAGE gels for electrophoresis using a PROTEIN II xi Cell system (Bio-Rad). The gels were then silver stained using Bio-Rad Silver Stain Plus kit reagents (Bio-Rad) according to the manufacturer's instructions. The silver-stained gels were scanned using a Densitometer GS-800 (Bio-Rad) and then analyzed using PDQuest software (Bio-Rad). Protein samples from 3 independent experiments, with each including 4 groups of control, SCU, HR, and SCU + HR, were analyzed by 2-DE. And, for each protein sample, triplicate electrophoreses were performed to ensure reproducibility. Comparisons were made between gel images of protein profiles obtained from control group versus HR group, control group versus SCU group, and HR group versus SCU + HR group. Quantitative analysis was performed using the Student's *t*-test and significantly differentially expressed protein spots (*p* < 0.05) with 1.5-fold or more increased or decreased intensity between different groups were selected and subjected to further identification by MALDI-TOF MS/MS. MS/MS analysis was performed in Institutes of Biomedical Sciences, Fudan University, Shanghai, China, as described before [12–14]. The PMF and MS/MS data collected were submitted as a combined search to MASCOT (Matrix Science, London, UK) using the GSP Explorer software, V3.5 (Applied Biosystems, Foster City, CA) against the NCBInr databases. The probability-based score, assuming that the observed match is significant (*p* < 0.05), had to be more than 64 when submitting PMF data to the database and had to be be more than 30 for individual peptide ions when submitting peptide sequence spectra.

### 2.5. Bioinformatic Analysis

Bioinformatic analysis was conducted similar to previous reports [15, 16]. Protein-protein interactions were obtained from the Search Tool for the Retrieval of Interacting Genes (STRING) database (http://string.embl.de/) which include direct (physical) and indirect (functional) associations derived from four sources: genomic context, high throughput, coexpression, and previous knowledge. For the 18 proteins found in the proteomic analysis, we focused on the greatest possible correlations (i.e., interactions with a score larger than 0.15) without any other proteins added by custom limit. Only direct interactions among the proteins were included in the interaction network.

### 2.6. Western Blotting Analysis

Western blotting assay was conducted as reported before [12–14]. In assay of protein levels of EIF6, HSPD1, and CCT6A, cells of control group, HR group, SCU group, or SCU + HR group were washed three times with cold TBS and then harvested using a cell scraper. Cells were lysed in 10 volume of cold lysis buffer (50 mM Tris-HCl, pH 7.2, 250 mM NaCl, 0.1% NP-40, 2 mM EDTA, 10% glycerol, 1 mM PMSF, 5 *μ*g/mL aprotinin, and 5 *μ*g/mL leupeptin) on ice. Lysates were centrifuged and then the supernatant protein was denatured by mixing with equal volume of 2 × sample loading buffer and then boiling at 100°C for 5 min. Then, an aliquot of 50 *μ*g protein was loaded onto a 12% SDS gel, separated electrophoretically, and transferred to a PVDF membrane (Bio-Rad). After the PVDF membrane was incubated with 10 mM TBS with 1.0% Tween 20 and 10% dehydrated skim milk to block nonspecific protein binding, the membrane was incubated with primary antibodies overnight at 4°C. The primary antibodies for HSPD1 and EIF6 were rabbit polyclonal anti-HSPD1 antibody (number 15282-1-AP, 1 : 1500) and rabbit polyclonal anti-EIF6 antibody (number 10291-1-AP, 1 : 1500), all from Proteintech Group, Inc. (Chicago, USA). The primary antibody for CCT6A was rabbit polyclonal anti-CCT6 antibody (number ab155541, 1 : 800) bought from Abcam company (Cambridge, USA). Blots were then incubated with HRP-conjugated goat anti-rabbit IgG (#7074, 1 : 500, Cell signaling company) for 2 h at room temperature and then visualized using chemiluminescence (Pierce Biotechnology, Rockford, IL).

### 2.7. Statistical Analysis

Data are given as the mean ± SD. For each variable, three independent experiments were carried out. Significances of difference between groups were determined by a nonpaired Student's *t*-test.

## 3. Results

### 3.1. Effects of SCU on Cell Viability under Normal Condition or HR Treatment

As shown in Figure 1, SCU at concentrations from 0.001 to 10 *μ*M exhibited no significant influence on cell viability after 24 h treatment (Figure 1(b)) or 48 h treatment (Figure 1(c)). SCU at 100 *μ*M exhibited cytotoxicity on cells. As shown in Figure 1(d), HR treatment caused decrease in cell viability while pretreatment of SCU at 1, 5, and 10 *μ*M could significantly inhibit decrease in cell viability induced by HR treatment. The results indicated the protective effects of SCU on cells against HR injury. Edaravone, used as positive control in the present experiment, also could inhibit the decrease in cell viability induced by HR treatment.

### 3.2. Proteomic Study of Protein Expression Profiles of Cells of Control Group, SCU Group, HR Group, and SCU + HR Group

**Figure 2:**
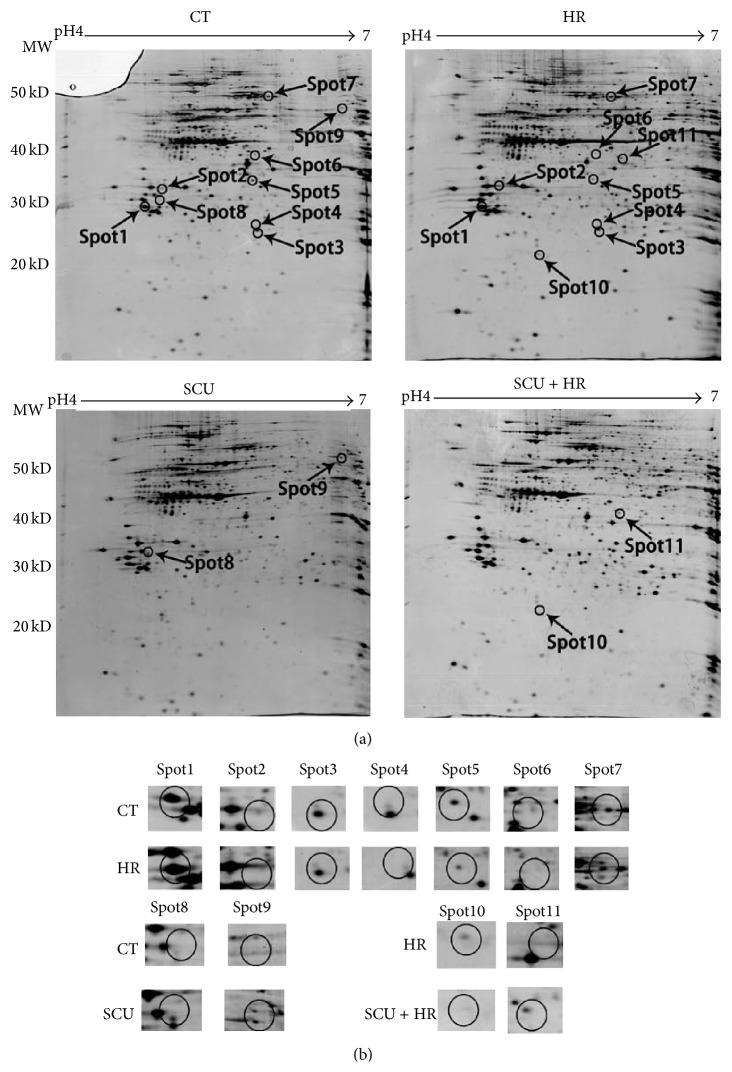
The proteome maps (2-DE images) of cytoplasm fraction of cells of control group, SCU group, HR group, and SCU + HR group. (a) Representative group of gels of nine replicate groups of gels, which were gotten by triplicate electrophoresis of samples from three independent experiments. Differentially expressed spots were shown by the arrows. (b) The expanded region of differentially expressed protein spots. The proteins within the circles were the differentially expressed proteins. Comparison was made between control group and HR group, control group and SCU group, and HR group and SCU + HR group.

**Figure 3:**
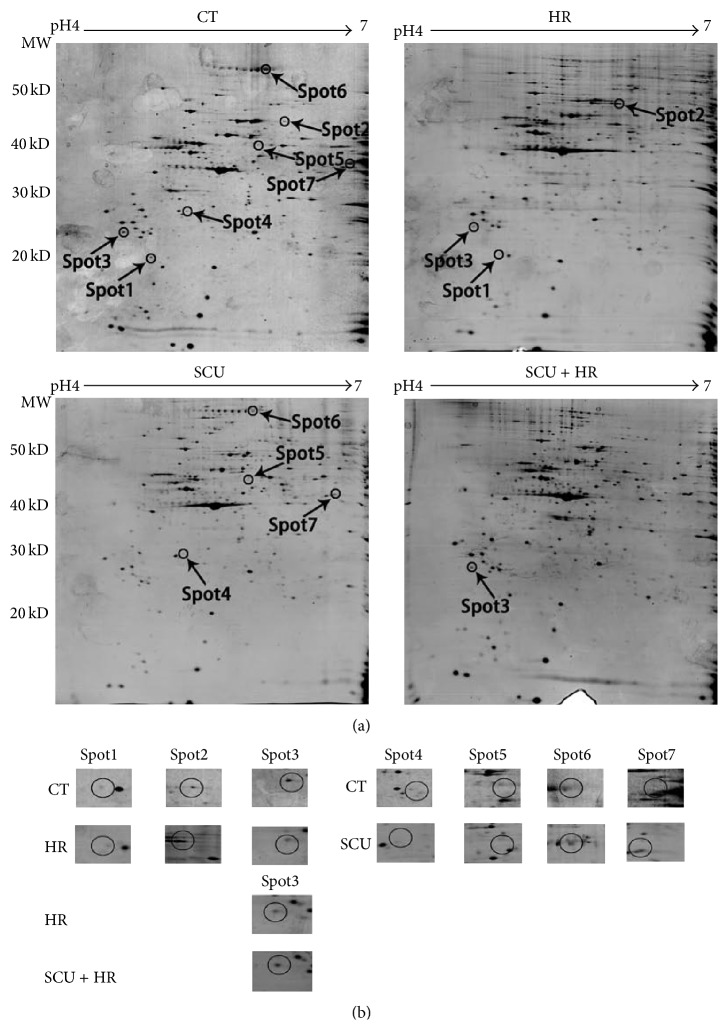
The proteome maps (2-DE images) of nuclei/membrane fraction of cells of control group, SCU group, HR group, and SCU + HR group. (a) Representative group of gels of nine replicate groups of gels, which were gotten by triplicate electrophoresis of samples from three independent experiments. Differentially expressed spots were shown by the arrows. (b) The expanded region of differentially expressed protein spots. The proteins within the circles were the differentially expressed proteins. Comparison was made between control group and HR group, control group and SCU group, and HR group and SCU + HR group.

**Figure 4:**
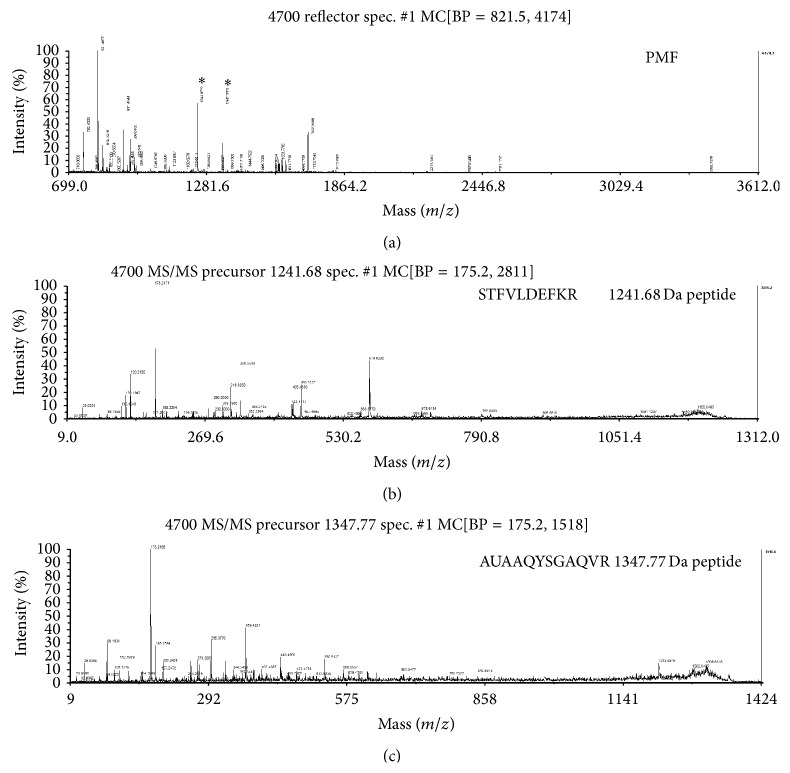
The result of the MALDI-TOF MS/MS analysis of spot7 cut from the 2-DE gels of nuclei/membrane fraction. It was identified to be human eukaryotic translation elongation factor 1 gamma by protein database search. (a) Peptide mass fingerprint of tryptic digest of the spot. *∗* unique peptides further identified by MS/MS. (b) MS/MS profile of the peptide with a mass of 11241.68 Da. (c) MS/MS profile of the peptide with a mass of 1347.77 Da.

To search possible target-related proteins of SCU related to its protective effects against HR injury, protein profiles of cells of control group, SCU group, HR group, and SCU + HR group were studied by comparative proteomic analysis. And, both cytoplasm fraction and nuclei/membrane fraction of the cells were analyzed. Representative 2D gel images of cytoplasm fraction and nuclei/membrane fraction were shown in Figures 2 and 3, respectively. Comparison was made between control group and HR group, control group and SCU group, and HR group and SCU + HR group. Significantly and differentially expressed protein spots (*p* < 0.05) with 1.5-fold or more increased or decreased intensity were accepted as differentially-expressed proteins. The protein spots were then cut from the gels, digested by trypsin, and identified using MS/MS. The result of MS/MS analysis of spot7 found in analysis of nuclei/membrane fraction was shown in Figure 4 as an example.

For cytoplasm fraction analysis (Figure 2), 11 differentially-expressed proteins were found totally, including 7 proteins found between control and HR, 2 proteins found between control and SCU, and another 2 proteins found between HR and SCU + HR group.  Table 1 showed both the information of these 11 protein spots such as the average intensity values and their standard deviations of the proteins spots and the fold differences between different groups as well as the MS/MS identification results of these proteins such as the protein score, sequence coverage, and best ion score of each spot.

For nuclei/membrane fraction analysis (Figure 3), 7 differentially-expressed proteins were found totally, including 3 proteins found between control and HR, 4 proteins found between control and SCU, and 1 protein found between HR and SCU + HR group. Notably, the differentially-expressed protein between HR and SCU + HR group, identified as p27BBP protein, was also found to be one of the differentially proteins between control and HR group.  Table 2 showed both the information of these 7 protein spots such as the average intensity values and their standard deviations of the proteins spots and the fold differences between different groups as well as the MS/MS identification results of these proteins such as the protein score, sequence coverage, and best ion score of each spot.

### 3.3. Possible Interaction Network of the Differentially-Expressed Proteins

**Figure 5:**
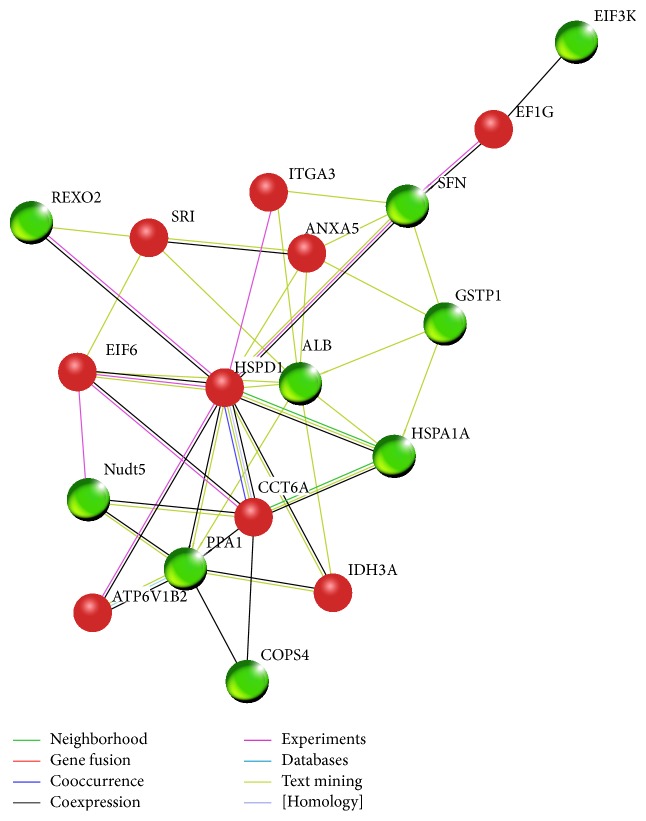
Protein-protein interaction network obtained from bioinformatic analysis based on STRING database. Possible target-related proteins of SCU were shown as round balls in red while possible proteins involved in HR injury were shown as round balls in green. Possible interactions between proteins were shown in line with different colors, as indicated in the figure.

The differentially-expressed proteins found in both comparison of control and SCU group and comparison of HR and SCU + HR group could be considered as possible target-related proteins of SCU. While the differentially-expressed proteins found in comparison of control and HR group might be important proteins involved in HR injury of cells. To elucidate possible mechanism of protective effects of SCU against HR injury, interaction network including both target-related proteins of SCU and proteins involved in HR injury was predicted using bioinformatic analysis. The information about abbreviated gene names and their corresponding protein full names was shown in Supplementary Table 1 available online at http://dx.doi.org/10.1155/2015/278014. As shown in Figure 5, target-related proteins of SCU (showed as red balls) and proteins involved in HR injury (showed as green balls) could link together into one net through direct interaction, that is, without adding intermediate partner. Notably, HSPD1 (heat shock 60 kDa protein 1) was exhibited to be the protein that had the most numerous connections with other proteins in the network. The results suggested that HSPD1 might be a critical factor in the protective effects of SCU.

### 3.4. Confirmation of Differentially-Expressed Proteins by Western Blotting

**Figure 6:**
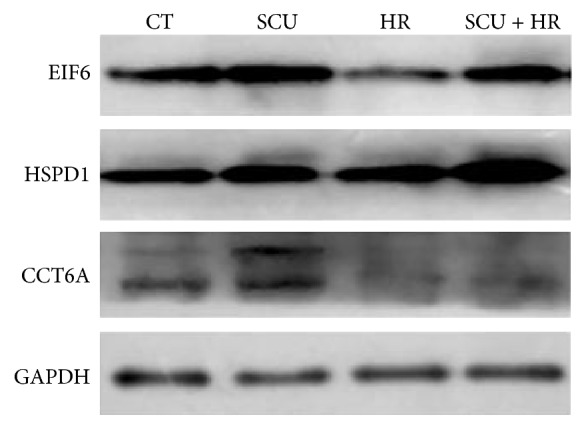
Results of Western blotting assay of protein levels of EIF6, HSPD1, and CCT6A in cells of control group, SCU group, HR group, and SCU + HR group shown were representative results of three independent experiments.

Western blotting was employed to assess the expression of EIF6, HSPD1, and CCT6A in cells of control and HR, SCU, and SCU + HR groups. As shown in Figure 6, expression level of EIF6 was decreased in HR group while SCU pretreatment (SCU + HR group) inhibited the decrease of EIF6 induced by HR injury. Expression levels of HSPD1 and CCT6A were increased in SCU group compared with control group. These results were consistent with the proteomic results.

## 4. Discussion

The present study demonstrated that SCU, a flavonoid glycoside successfully used in clinic in China for treatment of ischemic diseases, exhibited protective effects on HCMECs against HR injury. Previous reports had shown that SCU had protective effects on cardiomyocytes [6] and neurons [17] against hypoxia-related injury. Our results suggested that, in treatment of cardiac ischemia or brain ischemia, SCU might also exert protective effects on endothelium besides direct protective effects on cardiomyocytes or neurons. It was well known that cardiac ischemia injury could result in endothelium dysfunction [18, 19]. Damaged endothelium would reduce perfusion to areas of ischemia and thus exacerbated organ damage [20]. Furthermore, abnormalities in endothelial cell structure and function occurred earlier in ischemia-reperfusion injury than those in parenchyma cells, and the recovery of endothelial cells was later [21]. Since endothelial cells play a critical role in determining the extent of ischemia-reperfusion injury, protective effects of SCU on endothelial cells under injury might contribute to its efficiency in treatment of ischemic diseases.

Proteomic analysis of protein expression profiles of control cells, SCU-treated cells, HR-treated cells, and SCU + HR-treated cells in the present study provided information about both possible target-related proteins of SCU as well as proteins possibly involved in HR injury. The differentially-expressed proteins found in both comparison of control and SCU group and comparison of HR and SCU + HR group, with the total of 9 proteins including 4 found in cytoplasm fraction analysis and 5 found in nuclei/membrane fraction analysis, could be considered as possible target-related proteins of SCU. The differentially-expressed proteins found in comparison of control and HR group, with the total of 10 proteins including 7 found in cytoplasm fraction analysis and 3 found in nuclei/membrane fraction analysis, might be important proteins involved in HR injury of cells. Based on these results, possible interaction network including both target-related proteins of SCU and proteins involved in HR injury was predicted by bioinformatic analysis. Among these proteins, EIF6, HSPD1, and CCT6A might play important roles in the protective effects of SCU against HR injury.

EIF6, a monomeric protein of about 26 kDa, is an evolutionarily conserved protein involved in ribosomal subunit biosynthesis and assembly [22–24]. In the present study, EIF6 was found to be both a protein involved in HR injury and a possible target-related protein of SCU. In proteomic analysis, its expression level was downregulated in HR group compared with control while pretreatment of SCU could ameliorate the HR-induced decrease in EIF6 level. It was reported that EIF6 was a rate-limiting factor in translation, growth, and transformation [25, 26]. By binding to the large ribosomal subunit, EIF6 could prevent inappropriate interactions with the small subunit during initiation of protein synthesis [27]. Importantly, EIF6 might be part of a mechanism acting on the specific translation of messengers regulating cell survival such as regulating translation of factors upstream of Bcl2/Bax [28]. Previous reports showed that prolonged serum starvation could induce gradual decrease in EIF6 expression level [29]. Our finding that expression level of EIF6 was downregulated in HR-treated cells compared with control supported the role of EIF6 in cell survival. And, high level of EIF6 in SCU + HR-treated cells might protect cells against cell death induced by HR injury.

HSPD1 and CCT6A were found in the present study to be possible target-related protein of SCU whose expression levels were induced by SCU treatment. Interestingly, both proteins were chaperonin proteins. HSPD1 is a highly conserved protein which facilitates folding of nascent proteins into proper conformations thus plays a critical role in the assembly, folding, and transport of proteins in the mitochondria [30]. Mutations in HSPD1 were associated with diseases of nervous system [31, 32] and inactivation of HSPD1 in mice was embryonically lethal [33]. Furthermore, under heat stress, HSPD1 could interact with nascent cellular proteins to prevent their denaturation [30]. In the present study, HSPD1 was found to be located at the center of the interaction network and have the most numerous connections with other proteins in the network. Increase in HSPD1 in SCU-treated cells might protect proteins from HR injury thus increase viability of cells. Our results were consistent with previous reports that the overexpression of heat shock proteins could protect cells against apoptosis induced by ischemia-reperfusion injury [34–36]. CCT6A is the zeta subunit of the chaperonin containing TCP1 complex and is the only cytosolic chaperonin in eukaryotes. It was estimated to assist in the folding of multiple proteins such as actin, tubulin, cyclin E, myosin, and transducin [37]. Our finding about the increase levels of HSPD1 and CCT6A in SCU-treated cells suggested that induction of expression of chaperonin proteins might be involved in the protective effects of SCU against HR injury.

In the present study, HSPD1 and CCT6A were found to be a possible target-related protein of SCU in proteomic analysis of cytoplasm fraction and EIF6 was found to be a possible target-related protein of SCU in analysis of nuclei/membrane fraction. Our results were consistent with the reported subcellular location of HSPD1 in mitochondria [38, 39], CCT6A in cytoplasm [37], and nucleocytoplasmic shuttling of EIF6 [40, 41]. Interestingly, as shown in the network of bioinformatic analysis, EIF6 had direct interactions with HSPD1 and CCT6A. Therefore, it is possible that SCU might induce expression of chaperonin proteins, protect EIF6 in HR injury, keep translation of factors important in cell survival, and thus protect cells against HR injury. The roles of EIF6, HSPD1, and CCT6A in the protective effects of SCU against HR injury deserve further study.

In all, in the present study, the protective effects of SCU on HCMECs that underwent HR treatment were checked and possible target-related proteins of SCU were searched using proteomic analysis and analyzed by bioinformatic analysis. Results of the present study suggested possible important roles of EIF6, HSPD1, and CCT6A in the effects of SCU.

## Supplementary Material

The Supplementary table 1 provided the information of abbreviated genes names and their corresponding protein full names of proteins showed in Figure 5 of the manuscript. Figure 5 was the protein-protein interaction network obtained from bioinformatic analysis based on STRING database.

## Figures and Tables

**Table 1:** Differentially-expressed proteins found in proteomic analysis of cytoplasm protein expression profiles of different groups and results of MS/MS identification.

Spot	Target protein name	Accession number of NCBI database	Theoretical Mr (Da)/pI	Protein score	Sequence coverage (%)	Best ion score	Spot volume (ppm)		Fold change
							CT group	HR group	HR/CT

1	Chain A, 14-3-3 sigma in complex with Yap Ps127-peptide	306991738	26501/4.85	236	41	68	333.96 ± 223.4	172.3 ± 108.6	0.52

2	Chain B, crystal structure of human Nudt5 complexed with 8-oxo-Dgmp	317455160	21604/4.94	92	15	62	11.99 ± 14.9	1.36 ± 2.63	0.11

3	Glutathione S-transferase P	121746	23341/5.43	328	53	136	77 ± 71.7	27.6 ± 29.0	0.36

4	CGI-114 protein	5817183	26844/6.41	93	30	48	7.28 ± 6.2	2.4 ± 2.9	0.33

5	Inorganic pyrophosphatase	8247940	32639/5.54	179	34	66	36.88 ± 30.0	14.7 ± 8.3	0.40

6	COP9 complex subunit 4	5410300	46169/5.57	242	39	89	5.57 ± 4.79	2.26 ± 1.8	0.41

7	Heat shock protein	386785	69825/5.42	200	22%	59	52.9 ± 42.3	16.1 ± 17.9	0.30

							CT group	SCU group	SCU/CT

8	Heat shock 60 kDa protein 1 (chaperonin), isoform CRA_c	119590557	40896/5.09	73	11	58	7.17 ± 5.3	17.5 ± 11.6	2.44

9	Chaperonin containing TCP1, subunit 6A isoform variant	62089036	57725/6.25	224	22	56	1.76 ± 2.1	9.4 ± 8.4	5.34

							HR group	SCU + HR group	SCU + HR/HR

10	Sorcin isoform b	38679884	20332/5.11	98	31	64	5.3 ± 7.7	0.32 ± 0.58	0.06

11	Isocitrate dehydrogenase 3 (NAD+) alpha	18314368	39566/5.84	206	43	70	5.09 ± 4.26	10.13 ± 8.48	1.99

**Table 2:** Differentially-expressed proteins found in proteomic analysis of nuclei/membrane protein expression profiles of different groups and results of MS/MS identification.

Spot	Target protein name	Accession number of NCBI database	Theoretical Mr (Da)/pI	Protein score	Sequence coverage (%)	Best ion score	Spot volume (ppm)		Fold change
							CT group	HR group	HR/CT

1	Eukaryotic translation initiation factor 3, subunit 12, isoform CRA_b	119577208	17921/6.02	110	21	70	614.7 ± 423	182.1 ± 211.9	0.30

2	Albumin-like	763431	52048/5.69	138	9	86	831 ± 784	1997.7 ± 1131.4	2.41

3	p27BBP protein	13785574	26332/4.56	290	27	121	1868.7 ± 1220.8	726.1 ± 822.6	0.39

							CT group	SCU group	SCU/CT

4	Chain A, structural and electrophysiological analysis of annexin V mutants	157831406	35782/4.98	128	18	44	725.4 ± 314.8	241.7 ± 273.7	0.33

5	ATPase, H+ transporting, lysosomal 56/58 kDa, V1 subunit B2, isoform CRA_a	119584163	56465/5.57	87	15	31	584.1 ± 613.7	74.3 ± 126.8	0.13

6	VLA-3 alpha subunit	220141	113433/6.13	118	15	32	1812.1 ± 1377.5	536.9 ± 482.8	0.30

7	Eukaryotic translation elongation factor 1 gamma, isoform CRA_d	119594432	48407/6.24	187	28	47	4283.8 ± 2973.2	1790.3 ± 1335.2	0.42

							HR group	SCU + HR group	SCU + HR/HR

3	p27BBP protein	13785574	26332/4.56	290	27	121	726.1 ± 822.6	1797.3 ± 1085.5	2.48
