# Supplementary material for: Protective Effects of Scutellarin on Human Cardiac Microvascular Endothelial Cells against Hypoxia-Reoxygenation Injury and Its Possible Target-Related Proteins
Source: Evid Based Complement Alternat Med. 2015 Oct 18;2015:278014. doi: 10.1155/2015/278014 (PMC4628680; doi:10.1155/2015/278014)
Supplement: Supplementary file 1 — The Supplementary table 1 provided the information of abbreviated genes names and their corresponding protein full names of proteins showed in Figure 5 of the manuscript. Figure 5 was the protein-protein interaction network obtained from bioinformatic analysis based on STRING database. [file 278014.f1.pdf]

Supplementary Table 1. The information of abbreviated genes names and their corresponding protein full names of proteins showed in Figure 5

Abbreviated gene names (in alphabetical order)	Protein full names
ALB	albumin-like
ANXA5	Chain A, Structural And Electrophysiological Analysis Of Annexin V Mutants
ATP6V1B2	ATPase, H+ transporting, lysosomal 56/58kDa, V1 subunit B2, isoform CRA_a
CCT6A	chaperonin containing TCP1, subunit 6A isoform a variant
COPS4	COP9 complex subunit 4
EF1G	eukaryotic translation elongation factor 1 gamma, isoform CRA_d
EIF3K	eukaryotic translation initiation factor 3, subunit 12, isoform CRA_b
EIF6	p27BBP protein
GSTP1	Glutathione S-transferase P
HSPA1A	heat shock protein
HSPD1	heat shock 60kDa protein 1 (chaperonin), isoform CRA_c
IDH3A	Isocitrate dehydrogenase 3 (NAD+) alpha
ITGA3	VLA-3 alpha subunit

NUDT5	Chain B, Crystal Structure Of Human Nudt5 Complexed With 8-Oxo-Dgmp
PPA1	Inorganic pyrophosphatase
REXO2	CGI-114 protein
SFN	Chain A, 14-3-3 Sigma In Complex With Yap Ps127-Peptide
SRI	sorcin isoform b
